# AI-induced deskilling in orthopedic surgery: Preserving judgment in the age of intelligent decision support

**DOI:** 10.1051/sicotj/2026077

**Published:** 2026-10-07

**Authors:** Raju Vaishya, Abhishek Vaish

**Affiliations:** Department of Orthopaedics, Indraprastha Apollo Hospitals Sarita Vihar New Delhi 110076 India

**Keywords:** Artificial intelligence, Orthopedic surgery, Surgical education, Clinical decision-making, Automation bias

## Abstract

Artificial intelligence (AI) is rapidly entering orthopedic practice, offering efficiency gains while risking cognitive deskilling, particularly among trainees who may shift from generating clinical judgments to passively verifying algorithmic recommendations. Drawing on foundational automation-bias literature, cognitive-load theory, deliberate-practice principles, and Miller’s pyramid of competence, this editorial argues that orthopedic education must explicitly cultivate “AI independence” alongside AI fluency. We propose periodic AI-off assessments, a structured competency framework aligned with entrustable professional activities, and calibrated reliance modeled on aviation safety practices, so that future surgeons retain the capacity to reason, override, and remain accountable when intelligent systems are unavailable or incorrect.

## Editorial

Artificial intelligence (AI) is moving rapidly from an experimental technology to a component of everyday orthopedic practice. Its applications now include imaging interpretation, preoperative planning, clinical documentation, outcome prediction, patient communication, and decision support. The potential benefits are considerable. Yet an important question has received less attention than the capabilities of the technology itself: If AI increasingly performs the cognitive work of the surgeon, how do we ensure that surgeons continue to develop and maintain the ability to perform that work themselves?

The greatest threat of AI in orthopedics may not be that machines replace surgeons. It may be that surgeons gradually lose the ability to function without them. This concern is particularly relevant to training. A recent JBJS Open Access survey of orthopedic surgeons and trainees identified potential overreliance on AI and impairment of independent clinical judgment, particularly among less-experienced surgeons and trainees, as important concerns surrounding AI adoption [1].

Recent scoping and mixed-method reviews have synthesized emerging empirical evidence of AI-related deskilling across medicine. Heudel and colleagues [2] documented performance decrements when AI support was withdrawn and demonstrated automation bias even among experienced clinicians. Natali and colleagues [3] similarly highlighted erosion of core competencies (differential diagnosis, clinical judgment, and communication) and called for longitudinal monitoring and educational safeguards against “upskilling inhibition. These findings reinforce the need to treat deskilling as a measurable educational risk rather than a theoretical concern.

## From augmentation to cognitive substitution

Orthopedics has always embraced technology [4, 5]. Radiographs, computed tomography (CT), magnetic resonance imaging (MRI), navigation, and robotic systems have progressively enhanced what surgeons can see and do. AI differs in an important respect: rather than simply providing information or improving technical precision, it increasingly interprets information and proposes decisions:

*A navigation system can tell a surgeon where an implant is positioned. An AI system may tell the surgeon where it should be positioned.*

*A radiograph provides an image. An AI system may provide the diagnosis.*

*A clinical record contains information. A large language model may synthesize that information and recommend a management strategy.*

Not all AI applications represent cognitive substitution. Systems that organize information, highlight abnormalities, or reduce administrative burden primarily augment human cognition. The greater educational concern arises when AI generates diagnostic interpretations or management recommendations before independent analysis, effectively replacing rather than supporting key reasoning processes.

This transition from information augmentation to cognitive substitution creates a new educational problem. Expertise develops through repeated exposure to uncertainty, incomplete information, diagnostic error, and independent decision-making. If those cognitive steps are routinely outsourced, efficiency may improve while expertise quietly deteriorates. The concern is not hypothetical. Recent experimental evidence found that clinicians benefited from correct AI recommendations but became less accurate when the AI recommendation was incorrect, demonstrating AI’s potential to exert a substantial influence on clinical decisions [6].

Importantly, AI does not inevitably lead to deskilling. When used appropriately, AI may enhance learning by providing feedback, highlighting overlooked findings, and supporting pattern recognition. The educational challenge is therefore not whether AI should be used, but how it can be integrated in ways that preserve the trainee’s responsibility to generate independent clinical judgments before engaging with algorithmic recommendations.

## Historical roots of automation bias and the risk of deskilling

Concerns about overreliance on automated systems are not new. Parasuraman and Riley’s foundational 1997 analysis of human–automation interaction distinguished among use, misuse, disuse, and abuse of automation [7]. Misuse and overreliance produce both commission errors (following an incorrect automated recommendation) and omission errors (failing to act because the automation remains silent). Lee and colleagues [8, 9] subsequently refined the role of trust calibration, emphasizing that appropriate reliance requires operators to understand system capabilities and limitations rather than simply increasing trust.

A related phenomenon is the “lack-of-reasoning” or heuristic-replacement effect: operators may treat the automated output as a substitute for independent information search and analysis rather than as one input among many [10]. When the interface presents a fluent, coherent recommendation – particularly from a large language model – the cognitive effort of generating an alternative interpretation is reduced, and the probability of uncritical acceptance rises. This mechanism is especially relevant to generative AI, whose persuasive narrative form can mask underlying uncertainty or bias.

These dynamics have been observed across domains. In radiology and pathology, incorrect AI prompts measurably increase false-positive rates and reverse previously correct diagnoses [2]. In colonoscopy, adenoma detection rates have declined when endoscopists returned to unaided practice after periods of AI assistance [2]. The pattern is consistent with skill atrophy through reduced deliberate engagement with the underlying cognitive task.

## The trainee is a vulnerable point

The difference between an experienced surgeon and a trainee matters. An experienced surgeon can treat AI as a second opinion because years of clinical experience provide an internal framework against which the recommendation can be tested. A trainee may instead use the AI recommendation as the framework itself.

Consider a resident planning treatment for a complex periarticular fracture. Traditionally, the resident interprets the imaging, examines the patient, formulates a diagnosis, considers treatment alternatives, and proposes a plan before the attending challenges the reasoning. Imagine an AI system that immediately provides the fracture classification, identifies relevant anatomy, recommends fixation strategies, and predicts outcomes. The resident may become highly proficient at verifying an answer without learning how to generate one.

That distinction is fundamental. Surgical competence is not merely the ability to select the correct answer when it is presented. It is the ability to reason when the answer is not obvious, the data are incomplete, and the patient does not resemble the population from which the model learned. Table 1 summarizes the potential effects of increasing AI dependence across different domains of orthopedic training.

**Table 1:** Potential effects of increasing artificial intelligence (AI) dependence on orthopedic training.

Domain	Traditional training model	AI-dependent model	Potential consequence
Diagnosis	Trainee generates differential diagnosis independently	AI provides diagnosis early	Reduced diagnostic reasoning
Imaging interpretation	Trainee interprets images before discussion	AI-generated interpretation precedes trainee assessment	Automation bias
Treatment planning	Trainee develops and defends a plan	AI proposes management strategy	Reduced independent decision-making
Literature synthesis	Trainee searches, reads, and critically appraises evidence	AI summarizes evidence	Superficial appraisal and acceptance of errors
Operative planning	Surgeon mentally rehearses approach and alternatives	AI generates preferred approach	Reduced planning experience
Error recognition	Errors identified through experience and feedback	AI may identify or prevent errors	Reduced exposure to diagnostic and cognitive errors
Uncertainty management	Trainee learns to decide with incomplete information	AI provides probabilistic recommendations	Reduced tolerance for uncertainty
Clinical judgment	Built through repeated independent decisions	Increasingly supported by algorithmic recommendations	Potential cognitive deskilling

## Balancing cognitive load reduction with skill preservation

Cognitive load theory provides a useful lens for understanding both the benefits and the risks of AI [11]. Working memory is limited; intrinsic load (task complexity), extraneous load (poor instructional design or irrelevant information), and germane load (effort devoted to schema construction) compete for the same capacity. Well-designed AI can reduce extraneous load by organizing imaging findings, summarizing literature, or scaffolding differential diagnosis, freeing resources for germane processing and deeper learning. In high-volume or high-complexity settings, such support may improve efficiency and reduce fatigue-related error.

The risk arises when AI systematically removes the very cognitive operations that build expertise. If trainees never experience the full intrinsic load of generating a diagnosis or constructing a treatment plan under uncertainty, the germane load required for schema formation never occurs. Expertise, in Ericsson’s framework of deliberate practice, requires focused, repetitive engagement with tasks at the edge of current ability, accompanied by feedback and progressive refinement of mental representations [12, 13]. Routine cognitive substitution short-circuits this process. The educational challenge is therefore not to reject cognitive-load reduction, but to ensure that load reduction is selective and temporary rather than permanent and comprehensive.

A useful educational objective may be developing cognitive resilience: the ability to maintain safe and effective clinical reasoning when decision-support systems are unavailable, inaccurate, or in conflict with clinical findings. Preserving this capability should be a central goal of AI-integrated surgical education.

## Grounding AI competency in established educational theory

Miller’s pyramid of clinical competence offers a natural structure for situating AI-related skills [14]. At the base (“knows”), trainees must understand AI capabilities, limitations, data biases, and failure modes. At the next level (“knows how”), they must be able to formulate an independent diagnostic and management plan and then critically compare it with an AI recommendation. “Shows how” is assessed in controlled settings – simulations, OSCE stations, or structured oral examinations – where the trainee demonstrates the ability to override or accept AI advice with explicit justification. The apex (“does”) requires demonstration of calibrated, accountable AI use (and AI-independent performance) in the workplace. Contemporary extensions of the pyramid that emphasize entrustment and professional identity formation further reinforce that the ultimate goal is a surgeon who remains responsible and capable whether or not AI is available.

These expectations align with competency-based medical education and the ACGME milestone/entrustable professional activity (EPA) framework. Independent clinical reasoning and the ability to manage uncertainty are already core expectations; AI independence simply makes explicit a competency that has always been implicit. The proposed “AI-off” assessments mirror existing high-stakes tools: closed-book oral boards, OSCE stations that withhold decision-support aids, and workplace-based assessments that evaluate entrustment under realistic conditions.

## AI Independence should become a surgical competency

Orthopedic training programs traditionally evaluate knowledge, technical ability, communication, professionalism, and clinical judgment. In the AI era, another competency should be explicitly assessed: AI-independent clinical reasoning.

Trainees should periodically be required to make diagnostic and management decisions without AI assistance. These assessments should determine whether the trainee can:

1. Formulate an independent diagnosis before viewing an AI recommendation.
2. Develop a treatment plan without algorithmic assistance.
3. Identify when an AI recommendation conflicts with clinical findings.
4. Explain why an AI-generated recommendation may be incorrect.
5. Recognize hallucinations, dataset limitations, and algorithmic bias.
6. Communicate an independent clinical rationale to the patient.
7. Override an AI recommendation when clinical judgment warrants doing so.

Table 2 presents a proposed framework for assessing these competencies during orthopedic training. These principles are consistent with current recommendations for responsible integration of AI into surgical education, in which AI should complement rather than replace development of clinical reasoning and professional competencies [15].

**Table 2:** Proposed framework for artificial intelligence (AI) competency in orthopedic training.

Competency	Expected trainee behavior	Suggested assessment
Independent reasoning	Formulates a diagnosis and management plan before accessing AI	AI-off case assessment
AI literacy	Understands capabilities, limitations, and appropriate indications	Structured AI-competency examination
Critical appraisal	Identifies errors, hallucinations, bias, and inappropriate recommendations	AI-versus-expert case comparison
Calibrated trust	Accepts appropriate recommendations and challenges inappropriate ones	Discordant AI–expert scenarios
Override capability	Recognizes when clinical findings should supersede AI	Simulation or oral examination
Accountability	Remains responsible for the final clinical decision	Case-based assessment
AI-free practice	Demonstrates safe clinical reasoning without AI assistance	Periodic “AI-off” assessment
Reflective practice	Explains how AI changed, or did not change, the final decision	Portfolio or documented case reflection

## Lessons from aviation: Maintaining manual competence

Aviation provides a well-studied parallel. Pilots rely extensively on automation, yet training and regulatory policy deliberately preserve the ability to fly manually and to detect automation failure. The Federal Aviation Administration has issued multiple Safety Alerts for Operators (SAFOs) and Advisory Circulars emphasizing that pilots must maintain manual flight-path management skills, remain mentally “in the loop,” and perform continuous reasonableness checks on automated outputs [16–18]. Overreliance and mode confusion have been implicated in accidents; consequently, operators are expected to practice manual operations and to treat automation as a tool that requires active supervision rather than passive trust.

Orthopedic training should adopt a similar philosophy. Automation is integrated while manual (AI-independent) competence is deliberately maintained and periodically assessed. The goal is not distrust of AI but calibrated reliance by knowing when the system is likely to be reliable and when human judgment must predominate.

## The “AI-Off” test

One practical approach is to incorporate deliberate AI-free periods into surgical education. During selected case conferences, examinations, trauma assessments, and operative-planning exercises, trainees first formulate an independent assessment. AI is then introduced as a comparator. The trainee is required to answer a simple question: What did the AI add, and where might the AI be wrong? This transforms AI from an answer-generating tool into a cognitive challenge and creates an educational metric focused on justification rather than mere agreement.

The educational sequence should therefore be:

- *Independent reasoning → AI recommendation → comparison → critical appraisal → final decision.*

This is fundamentally different from:

- *AI recommendation → verification → acceptance.*

The former develops expertise through deliberate practice. The latter risks developing dependence. Educationally, the objective should not be agreement with AI but justification of clinical reasoning. A trainee who appropriately disagrees with an algorithm may demonstrate greater competence than one who correctly agrees without understanding the rationale.

## The surgeon remains accountable

Although trainees are particularly vulnerable to skill-development deficits, experienced surgeons are not immune to deskilling. Prolonged reliance on automated recommendations may reduce diagnostic vigilance, critical reassessment, and engagement with complex clinical uncertainty even among highly experienced practitioners.

Introducing AI does not transfer clinical responsibility from the surgeon to the algorithm. If an AI system recommends an inappropriate operation, misses a diagnosis, or generates a flawed treatment plan, the surgeon remains responsible for the decision. This accountability has important ethical, professional, and medicolegal implications. As AI becomes embedded in clinical workflows, surgeons may increasingly be expected to understand how recommendations were generated, document AI’s role in decision-making, and justify departures from either algorithmic advice or accepted standards of care. Increasing reliance on AI should therefore be accompanied by increasing (and not decreasing) understanding of its limitations.

Bias represents an additional concern because algorithmic performance can vary across patient populations and may reproduce or amplify disparities present in the underlying data [19]. The goal should therefore not be to increase surgeons’ trust in AI. The goal should be calibrated trust. That distinction may ultimately determine whether AI improves or undermines patient safety. The same principle of human accountability extends beyond clinical care to scientific communication. Recent orthopedic literature has demonstrated substantial use of AI-generated text in published articles, highlighting the need for greater transparency regarding AI use in scholarly work [20].

## A new principle for orthopedic education

The central question for orthopedic educators is no longer whether trainees should learn AI. They should. The more important question is what they must learn before they are allowed to rely on it. AI competency should therefore have two components:

A) *AI fluency:* knowing how to use AI effectively.
B) *AI independence:* knowing how to practice safely when AI is unavailable or wrong.

Together, AI fluency and AI independence form the foundation of cognitive resilience in modern surgical practice. Neither competency alone is sufficient; safe integration requires both effective AI use and the ability to function without it. AI independence proves more important than the first. As AI becomes increasingly capable, the comparative advantage of the surgeon will shift away from memorizing information toward contextual judgment, uncertainty management, communication, ethical reasoning, technical adaptability, and accountability. Those capabilities are unlikely to remain strong if they are rarely exercised.

## Conclusion

AI will become increasingly embedded in orthopedic surgery. The appropriate response is neither enthusiasm without scrutiny nor resistance to innovation; it is deliberate integration grounded in established principles of human – automation interaction and medical education. We should build systems in which AI handles tasks at which machines excel while surgeons retain and deliberately practice the cognitive skills for which human judgment remains essential. Cognitive-load reduction is a legitimate benefit, but it must not eliminate the deliberate practice required for expertise. The surgeon of the future should be able to say: “AI recommends this, and here is why I agree.” But equally important, that surgeon must be able to say: “AI recommends this, and here is why I disagree.” The latter may become a defining competency of the next generation of orthopedic surgeons. AI should make surgeons more capable, but not less necessary to themselves.

## Use of an AI tool

Grok AI was used to proofread the manuscript to improve the readability and English grammar. The final version was fully rechecked by the authors, who take complete responsibility for its contents.

## Data Availability

The search records and screening notes underlying this review are available from the corresponding author on reasonable request.
